# A homozygous missense variant in *CHRM3* associated with familial urinary bladder disease

**DOI:** 10.1111/cge.13631

**Published:** 2019-09-11

**Authors:** Glenda M. Beaman, Gabriella Galatà, Keng W. Teik, Jill E. Urquhart, Ali Aishah, James O'Sullivan, Sanjeev S. Bhaskar, Katherine A. Wood, Huw B. Thomas, Raymond T. O'Keefe, Adrian S. Woolf, Helen M. Stuart, William G. Newman

**Affiliations:** ^1^ Manchester Centre for Genomic Medicine Manchester University NHS Foundation Trust, Health Innovation Manchester Manchester UK; ^2^ Division of Evolution and Genomic Sciences, Faculty of Biology, Medicine and Human Sciences University of Manchester Manchester UK; ^3^ Department of Genetics Hospital Kuala Lumpur Kuala Lumpur Malaysia; ^4^ Division of Cell Matrix Biology and Regenerative Medicine, School of Biological Sciences, Faculty of Biology Medicine and Health University of Manchester, Manchester UK; ^5^ Royal Manchester Children's Hospital Manchester University NHS Foundation Trust, Manchester Academic Health Science Centre Manchester UK; ^6^ Peking University Health Sciences Center Beijing China

**Keywords:** *CHRM3*, distended bladder, prune belly, urinary bladder disease

## Abstract

*CHRM3* codes for the M3 muscarinic acetylcholine receptor that is located on the surface of smooth muscle cells of the detrusor, the muscle that effects urinary voiding. Previously, we reported brothers in a family affected by a congenital prune belly‐like syndrome with mydriasis due to homozygous *CHRM3* frameshift variants. In this study, we describe two sisters with bladders that failed to empty completely and pupils that failed to constrict fully in response to light, who are homozygous for the missense *CHRM3* variant c.352G > A; p.(Gly118Arg). Samples were not available for genotyping from their brother, who had a history of multiple urinary tract infections and underwent surgical bladder draining in the first year of life. He died at the age of 6 years. This is the first independent report of biallelic variants in *CHRM3* in a family with a rare serious bladder disorder associated with mydriasis and provides important evidence of this association.

## Graphical abstract

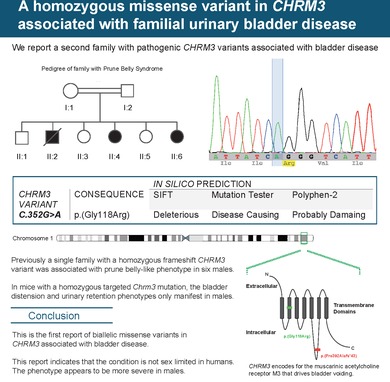

## 1 INTRODUCTION

Urinary bladder abnormalities not only devastate children's health but also cause major disruption to the lives of families. The last decade has witnessed discoveries of gene variants in specific classes of congenital urinary bladder abnormalities, including prune belly,^1^, ^2^ urofacial^3^, ^4^, ^5^, ^6^ and visceral myopathy (megacystis‐microcolon‐intestinal‐hypoperistalsis)^7^, ^8^, ^9^, ^10^, ^11^ syndromes. In some of these cases, the associated genes encode proteins involved in the intracellular contractile apparatus of bladder smooth muscle. These genes comprise *MYH11* encoding myosin light chain kinase,^8^ *ACTA2* encoding α‐smooth muscle actin,^2^ and *ACTG2* encoding γ2‐smooth muscle actin,^7^ all muscle cytoskeletal proteins; and *MYLK* that codes for myosin light chain kinase, required for myosin activation.^9^ In other cases, genes that have been associated with bladder malformations code for proteins implicated in the neuro‐muscular circuits required for bladder voiding.^12^, ^13^ As examples, *HPSE2* codes for heparanase 2 and *LRIG2* codes for leucine rich repeats and immunoglobulin‐like domains 2, proteins detected in foetal bladder nerves; variants of these genes are implicated in urofacial syndrome.^5^, ^6^, ^14^

Also in the neuro‐muscular category is *CHRM3* that encodes M3, the key acetylcholine receptor expressed by detrusor smooth muscle cells that is required for parasympathetic‐driven detrusor contraction and bladder emptying.^2^, ^13^, ^15^ Weber et al^1^ reported a family with six brothers who were born to consanguineous Turkish parents. They had congenital prune belly‐like syndrome characterised by hypocontractile bladders that failed to empty completely. Each affected boy available for testing harboured a homozygous frameshift variant of *CHRM3*, predicted to lead to a truncated protein and thus explaining the hypocontractile bladder phenotype. The affected brothers also had impaired pupillary constriction to light, a notable feature given that the M3 acetylcholine receptor is expressed in pupillary sphincter muscle, mediating its contractions.^15^, ^16^ Furthermore, male mice that carry homozygous targeted loss of function mutations of *Chrm3* have a similar syndrome affecting the bladder and the eye.^17^ In this report, we present a second family carrying a homozygous variant in *CHRM3* associated with familial urinary bladder disease.

## 2 METHODS

**Figure 1:**
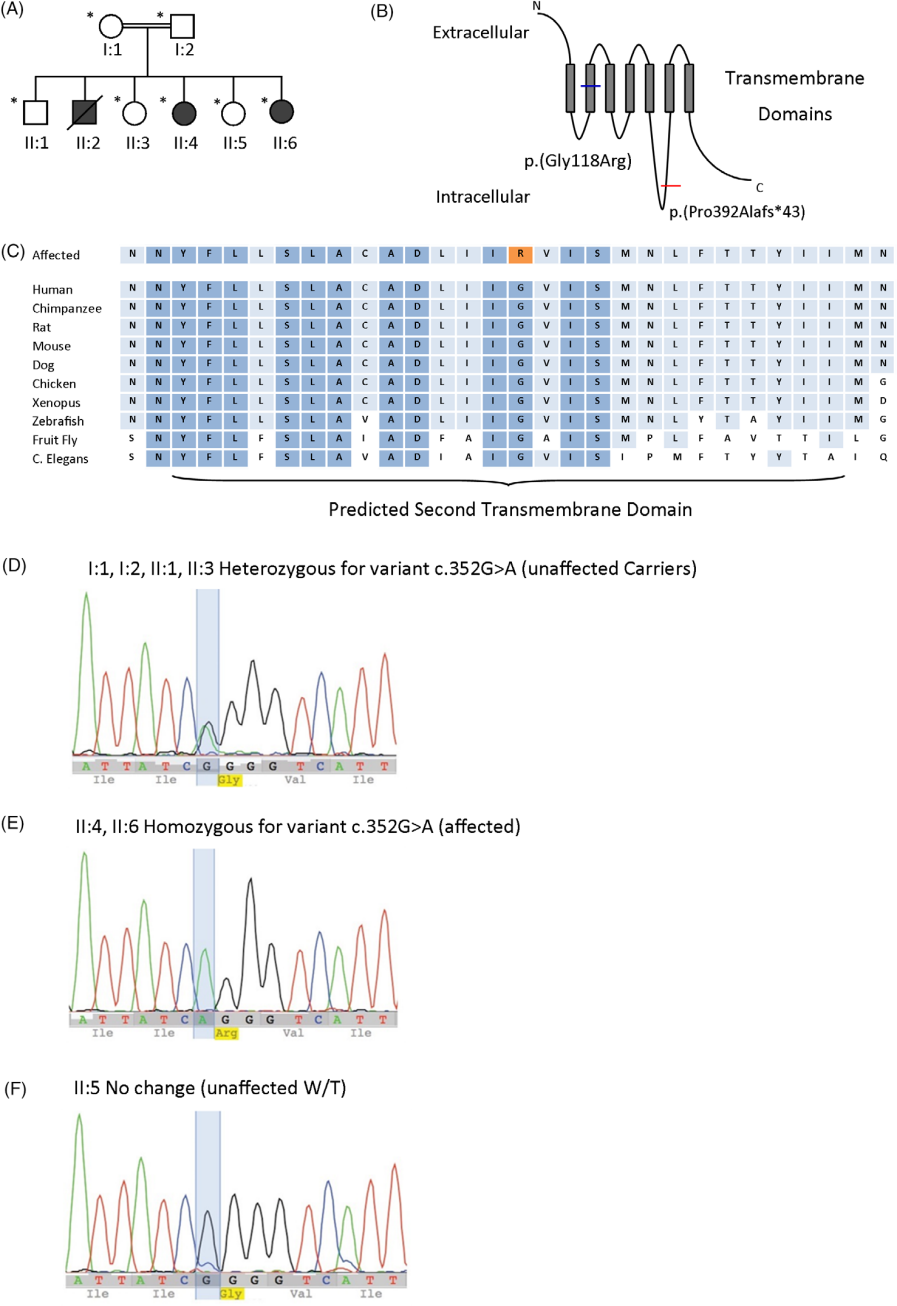
Identification of *CHRM3* mutation in Malaysian family. A, Family pedigree of Malaysian family, who were screened by Sanger sequencing for the c.352G > A variant, indicated by *. Filled in symbols represent affected individuals. B, Schematic diagram showing that the variant alters a residue within the second transmembrane domain of the M3 acetylcholine receptor and segregated with disease in the affected family. The diagram also depicts the frameshift variant identified by Weber et al.^1^ C, Predicted second transmembrane domain, the identified mutation alters a conserved glycine (G) residue which is highly conserved. D ‐ F, Genomic sequence chromatograms. ‐ D, I:1, I:2, II:1, II:3 Heterozygous for variant (unaffected carriers) (E) II:4, II:6 Homozygous for variant (affected) (F) II:5 Wild type (unaffected) [Colour figure can be viewed at http://wileyonlinelibrary.com]

The family (Figure 1A) was recruited as part of a nationally and internationally sourced cohort of 20 unrelated families with phenotypes overlapping urinary bladder voiding dysfunction of unknown cause. Independent of this case, we identified a single affected individual with a pathogenic de novo *ACTG2* c.769C > T, Arg257Cys variant. Institutional ethical review and approval (UK; University of Manchester [06138] and National Research Ethics Service North West, Greater Manchester Central ethics committee [06/Q1406/52 and 11/NW/0021]) was granted, and informed consent was provided.

A targeted enrichment to sequence all the exons of RefSeq transcripts of 13 genes previously associated with urinary bladder voiding abnormalities was designed (*HPSE2*, *LRIG2*, *CHRM3*, *ACTG2*, *MYH11*, *ACTA2*, *DSTYK*, *HNF1B*, *ROBO2*, *TBX18*, *TNXB*, *TSHZ3*, *UPK3A*). Using manufacturers' protocols, DNA samples from peripheral blood were enriched by an Agilent SureSelect Custom Design target‐enrichment kit (Agilent, Santa Clara, California) and sequenced with the Illumina MiSeq (Illumina, San Diego, California). Subsequent sequence alignment, variant calling, annotation and filtering were performed as reported previously.^18^ Variants of interest were confirmed using Sanger sequencing following standard procedures.

An 840 bp fragment of *CHRM3* was amplified from patient or reference genomic DNA by polymerase chain reaction (PCR) and cloned into the SK3 plasmid (a derivative of the pSpliceExpress mini‐gene splice reporter vector, a gift from Stefan Stamm, Addgene #32485) using the NEbuilder method (New England Biolabs). Successfully cloned constructs were transformed into competent bacteria and candidate colonies were cultured then vector DNA isolated. Sequences of mini‐gene vector constructs were verified by Sanger sequencing (performed by Eurofins Genomics).

HEK293 cells were cultured overnight to 40% to 50% confluency in Dulbecco's modified Eagle's medium high‐glucose, DMEM (Sigma), supplemented with 10% foetal bovine serum (Sigma) in six well tissue culture‐treated plates at 37°C with 5% CO_2_. Cells were transiently transfected with 1 μg of mini‐gene vector using Lipofectamine LTX (Thermofisher Scientific) and the manufacturer's recommended protocol. Following 20 hours incubation at 37°C with 5% CO_2_, RNA was extracted using the ReliaPrep RNA extraction kit (Promega) which included a DNase digestion step. An equal amount of RNA for each sample was converted to cDNA using Supercript IV (ThermoFisher Scientific). cDNA was amplified using Phusion polymerase and specific mini‐gene primers (Forward 5′‐GCACCTTTGTGGTTCTCACT‐3′, Reverse 5′‐GGGCCTAGTTGCAGTAGTTCT‐3′). Finally, PCR products were separated on an agarose gel (1%) supplemented with SafeView nucleic acid stain (NBS Biologicals) and visualised under a blue‐light transilluminator. Where appropriate, bands of interest were extracted and purified using QIAquick gel extraction kit (Qiagen) and sequenced by Sanger sequencing (Eurofins Genomics) to confirm their identity.

## 3 RESULTS

The proband (II:4) was found to have a novel homozygous missense variant in *CHRM3*, c.352G > A; p.(Gly118Arg) NM_000740.3. This variant was not present in a database of exome and genome sequence data from >120 000 healthy individuals (GnomAD, http://gnomad.broadinstitute.org) and is predicted by in silico tools of variant function to be probably damaging by Polyphen‐2, deleterious by SIFT and disease causing by MutationTaster. The variant was not found to influence splicing of the *CHRM3* pre‐mRNA in a mini‐gene splicing assay. The glycine residue at position 118 in the protein is conserved to zebra fish indicating its evolutionary importance. The variant alters a residue within the second transmembrane domain of the M3 acetylcholine receptor and segregated with disease in the affected family (Figure 1B‐F) such that the two affected members who were available for testing were homozygous for the variant. No other putative pathogenic variants were identified in the sequence panel testing undertaken in this family.

II:4 was born at full term to consanguineous Malaysian parents, antenatal scans were not performed. From birth, she was noted to have a distended abdomen and a poor urinary stream and from the second month, she was established on clean intermittent bladder catheterization. She had undergone numerous radiological investigations in the first months after birth. Ultrasonography revealed two kidneys of normal lengths but with mild to moderate hydronephrosis and dilated upper ureter on the left side; the bladder was distended with a slightly thickened and irregular wall. A micturating cystourethrogram found no evidence of spina bifida, urethral obstruction or vesicoureteral reflux but the bladder was enlarged and irregular, with a large residual volume. A Tc99m‐diethylenetriaminepentacetate (DTPA) isotope scan showed symmetrical function with tracer hold‐up in the left renal pelvis with wash‐out following intravenous furosemide diuretic administration. Magnetic resonance imaging of the lumbo‐sacral spine was normal. Urodynamics undertaken when she was 3 years showed a hypocontractile bladder with a normal capacity of 152 mL and at 11 years showed a functionally obstructed bladder with a normal capacity of 400 mL. During the first 3 years of life, she had one or two urinary tract infections each year and then no further infections until she was a teenager. She suffers from constipation. She left school in early childhood due to poor attainment but there is no evidence of specific intellectual disability. Assessment at 15 years old revealed that her pupils were 5 mm in diameter, with no constriction to light, and her urinary bladder was palpable (Table 1).

**Table 1:** Clinical features in individuals with disorders associated with CHRM3

	Family 1 (Weber et al 2011)^1^						F2 (Pomper et al 2011)^19^ ^a^	F3 (This report)		
	Male	Male	Male	Male	Male	Male	Male	Male	Female	Female
Status	Deceased	Alive	Alive	Alive	Alive	Alive	Alive	Deceased	Alive	Alive
Abdominal wall distension	+	−	−	+	−	−	−	−	+	−
Incomplete bladder emptying after voiding	u/k	+	+	+	+	+	+	u/k	+	+
Other urinary tract phenotype	Renal failure urosepsis	−	−	−	−	Bladder diverticulae	Urinary stones, urosepsis	Urosepsis		
Pupillary restriction to light	+	+	+	+	+	+	+	+	+	+
Dry mouth	+	+	+	+	+	+	−	u/k	−	−
Other phenotype	−	−	−	−	−	−	Lean habitus	−	Constipation, mild ID	Constipation, mild ID

Abbreviations: NR, not reported; u/k, unknown, +, feature present, −, feature absent.

a No *CHRM3* variants identified.

She has a clinically unaffected brother (II:1) who was heterozygous for the variant. Her other older brother (II:2) had died at the age of 6 years after a short unspecified illness. He had a history of multiple urinary tract infections and a vesicostomy had been fashioned at 8 months of age. Further details of the individual are not available. Tissue was not available for genetic testing. Her older clinically unaffected sister (II:3) was also heterozygous for the variant.

The index case has two younger siblings, both female. One (II.5) is alive and well with no urinary tract symptoms, and wild type for the variant. The other (II:6) had an antenatal ultrasound scan which revealed a distended bladder with left‐sided hydronephrosis. She was born at term and underwent several radiological investigations in the first 4 months of life. Ultrasonography showed a normal spine, two kidneys of normal length, bilateral hydronephrosis, right hydroureter and bladder wall thickening. Tc99m‐DTPA scanning showed that the right kidney contributed only 35% of total function and there was progressive tracer accumulation in the pelvicalyceal system that washed out adequately into the dilated ureter. A micturating cystourethrogram showed an irregular bladder outline, in keeping with a neurogenic bladder but there was no vesicoureteral reflux. She was treated with intermittent catheterisation. In the first 4 years of life, she had one to two urinary tract infections per year, despite antibiotic prophylaxis. On examination aged 6 years, she was noted to have a palpable urinary bladder. An ultrasound examination showed two kidneys of normal length and echogenicity. She suffered from intermittent constipation and at 9 years old requires special educational support for mild intellectual disability. She did not have a formal neurocognitive evaluation. Her pupils were 5 mm in diameter that constricted minimally to light.

## 4 DISCUSSION

Prune belly syndrome due to biallelic variants in *CHRM3* is an ultra‐rare disorder. No subsequent reports have been published since the original family in 2011.^1^ This report represents a second family with bladder disease in individuals carrying a homozygous *CHRM3* variant. In common with the family reported by Weber et al,^1^ the affected members in the kindred presented with dysfunctional voiding associated with hypocontractile bladders; moreover, in both families, the disease was present at birth. The current report also emphasises that the *CHRM3*‐associated disease is not sex‐limited because, while all clinically affected siblings in the Weber et al 1 report were male, the disease in the family presented here affected two girls and most likely also their brother who died in childhood. In contrast, in mice carrying a homozygous targeted *Chrm3* mutation, the overdistended bladder phenotype only manifests in males.^17^ This observation might be in part explained by the fact that the male urethra is longer than the female counterpart, so higher intra‐vesical pressures are needed to expel urine. In fact, the male phenotype may be more severe in humans, with early deaths only observed in males in this report and that of Weber et al.^1^ The precise mechanism whereby males are more severely affected is as yet unclear but may be related to the anatomical differences, including urethral length. In both the current family and the one reported by Weber et al,^1^ affected members had impairment of pupillary constriction in response to light. Indeed, the presence of dysfunction of both the bladder and the eye is fully consistent with the hypothesis that M3 function was absent, or severely impaired, in both families. While Weber et al^1^ described a homozygous frameshift variant, the current family harboured a homozygous missense variant (Figure 1B). Of interest, Pomper et al^19^ described a patient who had impaired micturition and pupillary constriction and also had decreased levels of M3 protein in bladder tissue; however, *CHRM3* sequencing and copy number analyses were normal. We speculate that this patient may have harboured non‐coding variants that affect transcription of *CHRM3*.

*CHRM3* comprises five exons although only one of these is coding. Therefore, the frameshift variant identified in the original report^1^ is likely to escape nonsense‐mediated decay and a truncated protein will likely be formed. The missense variant reported here has no effect on splicing in an in vitro system and although it is predicted to result in reduced function, the specific mechanism requires further elucidation.

Unfortunately, *CHRM3* genotyping data representing a healthy Malaysian population is not available either publically or could be generated through this study and so it is possible that p.(Gly118Arg) represents a rare polymorphism in this population. Other in silico evidence of conservation and predicted pathogenicity and segregation with the phenotype support the causality of this variant. Although the missense variant is absent in GnomAD, it is present as a somatic variant in a number of databases of genotypes in cancer tissues.

Heterozygous copy number variants at chromosome 1q43, the locus for *CHRM3*, have been reported associated with developmental problems. A 473 kb deletion, removing only *CHRM3*, was identified in a young male with autistic features.^20^ Parental samples were not available to determine whether this was de novo. Furthermore, a larger de novo deletion encompassing three genes, including *CHRM3* was present in a male with learning disability, cryptorchidism, short stature and alopecia.^21^ A 763 kb duplication encompassing *CHRM3* alone was detected in a male with intellectual and developmental delay, autistic behaviour, short stature, and hand anomalies.^22^ It is important to note that the parents in the original report and in our family with heterozygous *CHRM3* variants are clinically normal. Therefore, these data indicate that dosage effects of *CHRM3* may be associated with a clinical developmental phenotype, distinct from the bladder and pupillary phenotype in individuals with biallelic coding variants.

*CHRM3* is overexpressed in bladders of adults with benign prostatic hypertrophy,^20^ most likely a compensatory response to anatomic bladder outflow obstruction. The M3 acetylcholine receptor is in fact more widely expressed than in the detrusor and ciliary muscles.

Although dilated pupils are present in all affected individuals reported to date with biallelic *CHRM3* variants indicating that this is a core feature of the phenotype, the association of bladder dysfunction with mydriasis is not exclusive to individuals with *CHRM3* variants. Of note individuals with biallelic pathogenic variants in *MYL9* have mydriasis as part of megacystis microcolon intestinal hypoperistalsis phenotype.^11^ The similarity in the bladder‐eye phenotype in the two families would indicate that the missense variant results in a loss of function comparable to that predicted by the frameshift variant identified by Weber et al.^1^ It will be important through murine and cellular studies to undertake functional studies to determine the mechanism of action of the reported disease‐associated variants. The report of this second family provides independent evidence that biallelic variants in *CHRM3* result in severe bladder voiding dysfunction which on the basis of the families described to date is associated with mydriasis and is more severe, resulting in early lethality, in males.

## CONFLICT OF INTEREST

Nothing to declare.

## Data Availability

The data that support the findings of this study are available from the corresponding author upon reasonable request.

## References

[1] Weber S , Thiele H , Mir S , et al. Muscarinic acetylcholine receptor M3 mutation causes urinary bladder disease and a prune‐belly‐like syndrome. Am J Hum Genet. 2011;89:668‐674. 22077972 10.1016/j.ajhg.2011.10.007 PMC3213389

[2] Richer J , Milewicz DM , Gow R , et al. R179H mutation in ACTA2 expanding the phenotype to include prune‐belly sequence and skin manifestations. Am J Med Genet A. 2012;158A:664‐668. 22302747 10.1002/ajmg.a.35206

[3] Daly SB , Urquhart JE , Hilton E , et al. Mutations in *HPSE2* cause urofacial syndrome. Am J Hum Genet. 2010;11:963‐969. 10.1016/j.ajhg.2010.05.006 PMC3032078 20560210

[4] Pang J , Zhang S , Yang P , et al. Loss‐of‐function mutations in HPSE2 cause the autosomal recessive urofacial syndrome. Am J Hum Genet. 2010;86:957‐962. 20560209 10.1016/j.ajhg.2010.04.016 PMC3032074

[5] Stuart HM , Roberts NA , Bergu B , et al. *LRIG2* mutations cause urofacial syndrome. Am J Hum Genet. 2013;92:259‐264. 23313374 10.1016/j.ajhg.2012.12.002 PMC3567269

[6] Stuart HM , Roberts NA , Hilton EN , et al. Urinary tract effects of *HPSE2* mutations. J Am Soc Nephrol. 2015;26:797‐804. 25145936 10.1681/ASN.2013090961 PMC4378092

[7] Thorson W , Diaz‐Horta O , Foster J 2nd , et al. De novo ACTG2 mutations cause congenital distended bladder, microcolon, and intestinal hypoperistalsis. Hum Genet. 2014;133:737‐742. 24337657 10.1007/s00439-013-1406-0

[8] Gauthier J , Ouled Amar Bencheikh B , Hamdan FF , et al. A homozygous loss‐of‐function variant in MYH11 in a case with megacystis‐microcolon‐intestinal hypoperistalsis syndrome. Eur J Hum Genet. 2015;23:1266‐1268. 25407000 10.1038/ejhg.2014.256 PMC4538215

[9] Halim D , Brosens E , Muller F , et al. Loss‐of‐function variants in MYLK cause recessive megacystis microcolon intestinal hypoperistalsis syndrome. Am J Hum Genet. 2017;101:123‐129. 28602422 10.1016/j.ajhg.2017.05.011 PMC5501771

[10] Halim D , Wilson MP , Oliver D , et al. Loss of LMOD1 impairs smooth muscle cytocontractility and causes megacystis microcolon intestinal hypoperistalsis syndrome in humans and mice. Proc Natl Acad Sci U S A. 2017;114:E2739‐E2747. 28292896 10.1073/pnas.1620507114 PMC5380076

[11] Moreno CA , Sobreira N , Pugh E , et al. Homozygous deletion in MYL9 expands the molecular basis of megacystis‐microcolon‐intestinal hypoperistalsis syndrome. Eur J Hum Genet. 2018;26:669‐675. 29453416 10.1038/s41431-017-0055-5 PMC5945668

[12] Benarroch EE . Neural control of the bladder: recent advances and neurologic implications. Neurology. 2010;75:1839‐1846. 21079186 10.1212/WNL.0b013e3181fdabba

[13] Keast JR , Smith‐Anttila CJ , Osborne PB . Developing a functional urinary bladder: a neuronal context. Front Cell Dev Biol. 2015;3:53. 26389118 10.3389/fcell.2015.00053 PMC4555086

[14] Roberts NA , Hilton EN , Lopes FM , et al. Lrig2 and Hpse2, mutated in urofacial syndrome, pattern nerves in the urinary bladder. Kidney Int. 2019;95:1138‐1152. 30885509 10.1016/j.kint.2018.11.040 PMC6481288

[15] Choppin A , Eglen RM , Hegde SS . Pharmacological characterization of muscarinic receptors in rabbit isolated iris sphincter muscle and urinary bladder smooth muscle. Br J Pharmacol. 1998;124:883‐888. 9692772 10.1038/sj.bjp.0701920 PMC1565469

[16] Gil DW , Krauss HA , Bogardus AM , WoldeMussie E . Muscarinic receptor subtypes in human iris‐ciliary body measured by immunoprecipitation. Invest Ophthalmol Vis Sci. 1997;38:1434‐1442. 9191607

[17] Matsui M , Motomura D , Karasawa H , et al. Multiple functional defects in peripheral autonomic organs in mice lacking muscarinic acetylcholine receptor gene for the M3 subtype. Proc Natl Acad Sci U S A. 2000;97:9579‐9584. 10944224 10.1073/pnas.97.17.9579 PMC16907

[18] Ellingford JM , Barton S , Bhaskar S , et al. Molecular findings from 537 individuals with inherited retinal disease. J Med Genet. 2016;53:761‐767. 27208204 10.1136/jmedgenet-2016-103837 PMC5106339

[19] Pomper JK , Wilhelm H , Tayebati SK , et al. A novel clinical syndrome revealing a deficiency of the muscarinic M3 receptor. Neurology. 2011;76:451‐455. 21282591 10.1212/WNL.0b013e31820a0a75

[20] Petersen AK , Ahmad A , Shafiq M , Brown‐Kipphut B , Fong CT , Anwar IM . Deletion 1q43 encompassing only CHRM3 in a patient with autistic disorder. Eur J Med Genet. 2013;56:118‐122. 23253743 10.1016/j.ejmg.2012.11.003

[21] Perrone MD , Rocca MS , Bruno I , Faletra F , Pecile V , Gasparini P . De novo kb interstitial deletion on chromosome 1q43 in a boy with mental retardation and short stature. Eur J Med Genet. 2012;55:117‐119. 22186213 10.1016/j.ejmg.2011.11.004

[22] Cheng X , Yang Q , Liu J , et al. Constitutional 763.3 kb chromosome 1q43 duplication encompassing only CHRM3 gene identified by next generation sequencing (NGS) in a child with intellectual disability. Mol Cytogenet. 2019;12:16. 31019551 10.1186/s13039-019-0427-3 PMC6472087

